# Neodymium magnets migrated into an internal supravesical hernia: a rare case of foreign body ingestion in children

**DOI:** 10.1186/s40792-023-01713-9

**Published:** 2023-07-19

**Authors:** Michiaki Ikegami, Yuichiro Miyaki, Takashi Hamano, Yurina Taira, Toshiaki Takahashi

**Affiliations:** 1 Department of Pediatric Surgery, Seirei Hamamatsu General Hospital, 2-12-12 Sumiyoshi, Naka Ward, Hamamatsu City, Shizuoka 430-8558 Japan; 2 Department of Surgery, Seirei Hamamatsu General Hospital, Hamamatsu, Japan; 3 Department of Colorectal Surgery, Seirei Hamamatsu General Hospital, Hamamatsu, Japan

**Keywords:** Neodymium magnet, Foreign body ingestion, Children, Laparoscopic removal

## Abstract

**Background:**

Foreign body (FB) ingestion is a common event in children. The management of the ingested FB depends on the location, type, number, size of the FBs, patient age, and symptoms. Although most FBs pass spontaneously through the gastrointestinal tract without causing serious injuries, the ingestion of multiple high-powered magnetic pieces, especially neodymium magnets (NMs) increases the risks of morbidity and mortality. Supravesical hernia (SH) is rarely occurs in children, and few studies have reported SH in pediatric patients. We report an extremely rare case of ingested NMs that migrated into an internal SH in a pediatric patient.

**Case presentation:**

A 3-year-old boy who had accidentally swallowed two NMs 3 days ago presented with vomiting and lower abdominal pain. X-ray imaging and computed tomography (CT) suspected the presence of a 1.0-cm radiopaque FB located in the terminal ileum dorsal side of the bladder. Although his abdominal pain was gradually getting better after oral feeding, repeat abdominal X-ray imaging showed that the FB was in a stagnant in position. Therefore, surgical intervention was planned to remove the FB 1 week after his admission. Under general anesthesia, laparoscopic and fluoroscopic examinations were performed and the cecum was found adhered to the retroperitoneum between the right medial umbilical fold and the right wall of the urinary bladder. The FB was presumed to be located at the tip of the incarcerated cecum in the retroperitoneal space. Peritoneum incision was started near the medial inguinal fossa, and the Retzius space was opened in a manner similar to the transabdominal pre-peritoneal approach for inguinal hernia repair. Consequently, the patient was diagnosed with internal SH with FB migration. The incarcerated cecum was pulled out, which revealed intestinal wall perforation. The FB remained in the retroperitoneal space in the pelvic cavity. The FB was easily removed using intestinal forceps and identified as combined two NMs. The postoperative course was good, and the patient was discharged on postoperative day 5.

**Conclusions:**

We experienced an extremely rare case of a pediatric patient who swallowed multiple NMs that migrated into an internal SH, and the laparoscopic minimally invasive removal was successful.

## Background

Foreign body (FB) ingestion is a common event in children [1]. The management of FB ingestion depends on the location, kind, number, and size of the ingested FBs, and the age and symptoms of the patients. Most FBs pass spontaneously through the gastrointestinal tract without causing serious injuries [1, 2]. However, ingestion of multiple high-power magnet pieces increases the risks of surgical intervention. In particular, neodymium magnets (NMs), which have recently been reported to cause serious bowel injuries, can increase the risk of morbidity and mortality.

Supravesical hernia (SH) is one of the rare internal hernias that are sometimes found in men after the age of 50 years [3]. The internal type of SH, which remains within the abdomen passing into the space around the bladder, is extremely rare [4, 5].

Some studies have reported FB cases with intestinal hernia [6–8]. These reports have demonstrated the risk of perforation if a FB enters an inguinal hernia sac. However, no studies have reported the relationship between FB ingestion and internal SH.

In this report, we describe a rare case of ingested NMs that migrated into an internal SH in pediatric patient. The precise location of the NMs was identified with multiple examination modalities, and they were successfully removed by laparoscopic approach.

## Case presentation

**Fig. 1:**
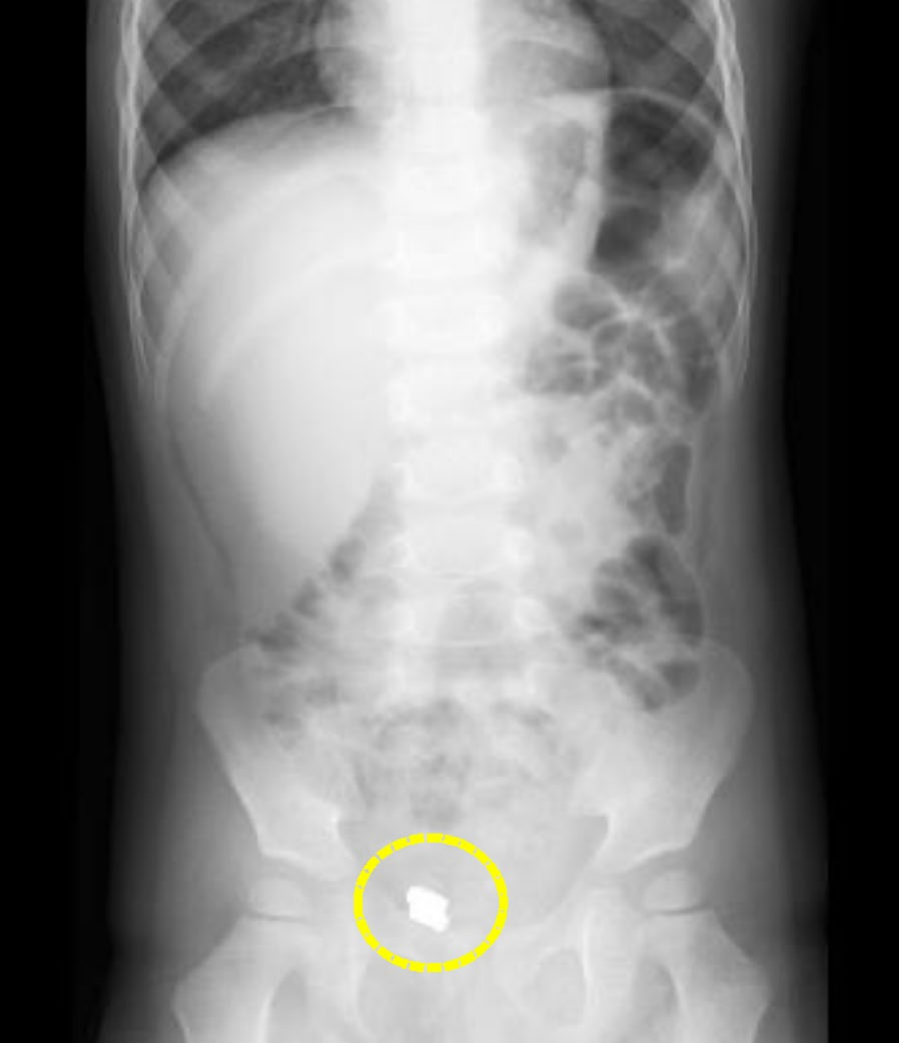
X-ray imaging. X-ray image revealed the presence of a 1.0-cm radiopaque FB (yellow circle) located in the right lower abdomen area on day 1. We expected that bowel movement might be able to evacuate the FB

**Fig. 2:**
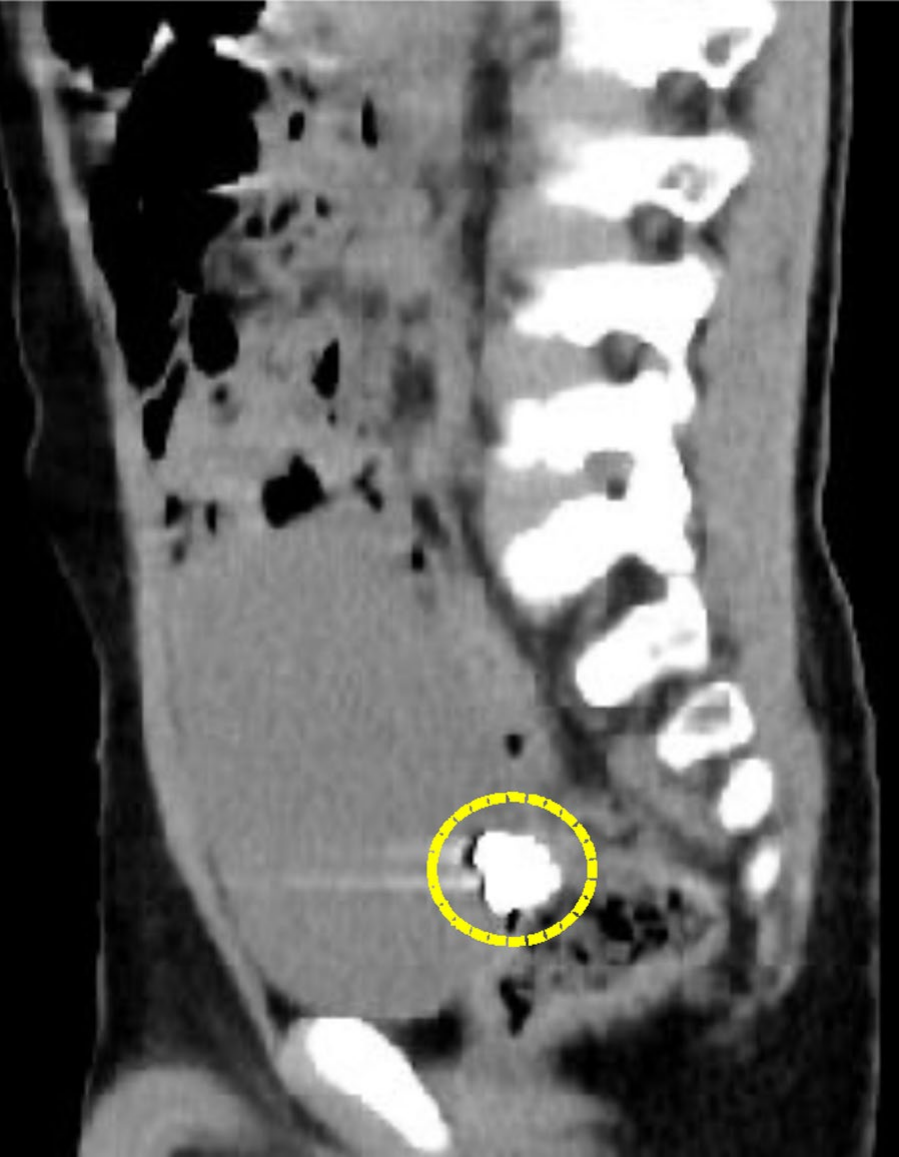
CT imaging (sagittal). On CT, the FB (yellow circle) was suspected to locate in the terminal ileum dorsal side to the bladder. There were no characteristic findings of SH that the bladder was suppressed by the incarcerated small bowel

A 3-year-old boy presented with vomiting and lower abdominal pain with a 3-day history of symptoms. He had no significant medical, surgical, or family history. On physical examination, he was afebrile with normal vital signs. Although he had tenderness localized to the right lower abdomen, his abdomen was soft without a rebound. Laboratory tests revealed high levels of inflammatory markers (white blood cell count of 15,760/μL and a C-reactive protein level of 1.64 mg/dL), with a normal lactate level. An X-ray imaging revealed the presence of a 1.0-cm radiopaque FB located in the right lower abdomen area (Fig. 1). According to his parents, he had accidentally swallowed two NMs 3 days ago. Computed tomography (CT) showed that the FB was suspected to be located in the terminal ileum dorsal side of the bladder (Fig. 2). When he had been transferred to our hospital, from the previous hospital, no evidence of free air or abscess formation was noted, and his abdominal pain was gradually getting better. Therefore, oral feeding was started, and a purgative medicine was administered to cause bowel movements and evacuate the FB. Although there was no recurrence of his symptoms and his condition was stable, a repeat abdominal X-ray imaging showed the FB was stagnant in position.

**Fig. 3:**
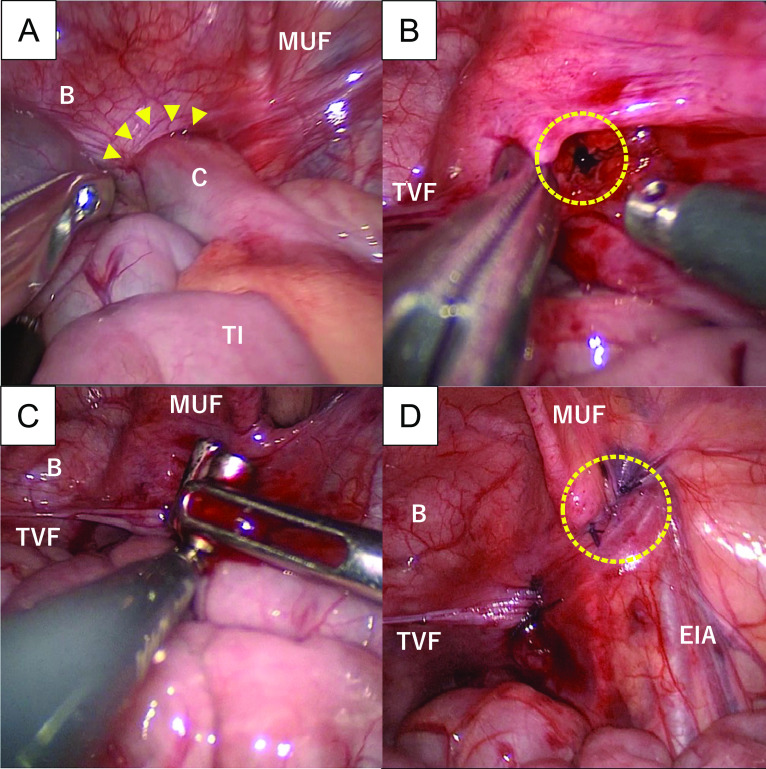
Laparoscopic findings. **A** The cecum adhered to the retroperitoneum between the right medial umbilical fold and the right wall of the urinary bladder (yellow arrowhead), making us suspect internal SH. **B** The FB (yellow arrowhead)remained in the retroperitoneal space in the pelvic cavity. **C** The FB was easily pulled out using intestinal forceps and identified as a combined two NMs. No intestinal tissue was found between the two magnetic bodies. **D** The perforated cecum was repaired from the umbilical wound by direct vision, and the retroperitoneal wall (yellow arrowhead) was repaired laparoscopically with absorbable sutures. *B* bladder, *C* cecum, *EIA* external iliac artery, *MUF* medial umbilical fold, *TI* terminal ileum, *TVF* transverse vesical fold

Therefore, surgical intervention was planned to remove the FB 1 week after his admission. Under general anesthesia, endoscopic examination for the total colon and terminal ileum region was initially performed under fluoroscopic guidance; however, we could not find the FB in his intestinal tract, so laparoscopic observation was then performed. After pneumoperitoneum induction, three trocars with a 5-mm diameter were placed into the single-incision laparoscopic surgical port at the umbilicus and a trocar into the right abdominal quadrant. Upon inspection, the cecum was found adhered to the retroperitoneum between the right medial umbilical fold and the right wall of the urinary bladder (Fig. 3A). The cecum could not be pulled out via traction or external manual compression, which made us suspect internal SH. Under fluoroscopic examination, the FB was presumed to be located at the tip of the incarcerated cecum in the retroperitoneal space. A peritoneum incision was started near the medial inguinal fossa, and the Retzius space was opened in a manner similar to the transabdominal pre-peritoneal (TAPP) approach for inguinal hernia repair. Then, the precise anatomical location of the incarcerated cecum was found, and the patient was diagnosed with internal SH with the FB migration.

The incarcerated cecum was very carefully pulled out from the retroperitoneum between the right medial umbilical fold and the right wall of the urinary bladder, which revealed the perforated intestinal wall. The FB remained in the retroperitoneal space in the pelvic cavity (Fig. 3B). The FB was easily removed using intestinal forceps and identified as a combined two NMs (Fig. 3C). No intestinal tissue was found between the two magnetic bodies. After abundant washing of the abdominopelvic cavity, the perforated cecum was repaired from the umbilical wound by direct vision and the retroperitoneal wall laparoscopically with absorbable sutures (Fig. 3D). Through a laparoscopic approach, the NMs inside the internal SH were successfully removed without injuring the bladder or other intraabdominal organs. The total operative time was totally 228 min. The postoperative course was good, and the patient was discharged on postoperative day 5.

## Discussion

Rare earth metals, such as neodymium, are used in highly powered magnets that are commonly used in toys, kitchen utensils, desk items, and many household products mainly because of their impressive strength-to-size ratio [1]. Its bounding strength of that is 5–30 times that of a conventional magnet [9]. Magnet pieces attract each other across the walls of the gastrointestinal tract, causing ischemia, tissue necrosis, perforation, fistula formation, obstruction, peritonitis, or death [10]. Recently, the incidence of magnet-related injuries has increased with the spread of NMs [2]. Lindley et al. presented of a case of an adolescent boy with intestinal obstruction secondary to multiple magnetic FB ingestion and sequestration within Meckel’s diverticulum [2]. Moreover, Camacho-Gomez et al. reported a case of an 11-year-old boy with ascending colon perforation secondary to the ingestion of a rare earth magnet [11].

Internal SH was first reported by Ring in 1814, and it is an extremely rare condition, particularly in children. SH occurs in the triangular space surrounded laterally by the medial umbilical ligament, medially by the median umbilical ligament (urachus), and inferiorly by the transverse vesical fold, and it is classified as external or internal depending on the direction of sac extension [4, 5, 12–14]. External SH protrudes through the anterior abdominal wall, expanding into the inguinal canal and mimicking a direct inguinal hernia. On the other side, an internal SH that remains within the abdomen passing into the space around the bladder is extremely rare and occurs mainly in men in their 50s and 60s [4, 5, 13]. The characteristic CT findings of SH is the bladder being suppressed by the incarcerated small bowel; however, an accurate preoperative diagnosis was made only in a few cases because SH is extremely rare [14]. The present case had no characteristic CT findings, and the accurate diagnosis could not be made preoperatively. Among the 38 cases of SH in the literature from 1982 to 2022, only one pediatric case was reported. Some studies have reported FB cases with intestinal hernia [6–8]. These studies have demonstrated a risk for perforation if the FB enters an inguinal hernia sac. However, no study has reported the relationship between FB ingestion and internal SH.

In this report, a 3-year-old boy accidentally swallowed two NMs 3 days ago and was admitted with vomiting and lower abdominal pain. CT showed that the FB was suspectedly located in the terminal ileum dorsal side of the bladder. However, by laparoscopic observation, the FB was found in the tip of the incarcerated cecum in the retroperitoneal space. Subsequently, the Retzius space was opened in a manner similar to the TAPP approach for inguinal hernia repair, which revealed the precise anatomical location of the FB in the incarcerated cecum; as a result, the diagnosis of SH with FB migration was made.

Lee et al. demonstrated his management strategy for FB ingestion in 2018. If multiple magnets or a single magnet with a metallic FB is located in sites beyond the stomach, symptomatic children must consult a pediatric surgeon to plan for surgery; however asymptomatic children may be closely followed using serial X-ray imaging to monitor the progression of the FBs [15]. In this case, according to his parent’s story, the patient had accidentally swallowed two NMs almost at once. The two NMs were speculated to be already be stuck each other while in the upper gastrointestinal tract because NM has extremely high magnetic power. In addition, CT showed that the FB was suspected to locate in the terminal ileum, and we expected that bowel movement might be able to evacuate the FB. Moreover, when he had been transferred to our hospital from the previous hospital, his slight abdominal pain was gradually getting better, and his symptoms disappeared within a few days. Even after oral feeding was initiated, the symptoms did not recur, and his condition was stable. Therefore, we monitored his condition very carefully and repeated abdominal X-ray imaging. However, surgical treatment could have been considered earlier.

**Fig. 4:**
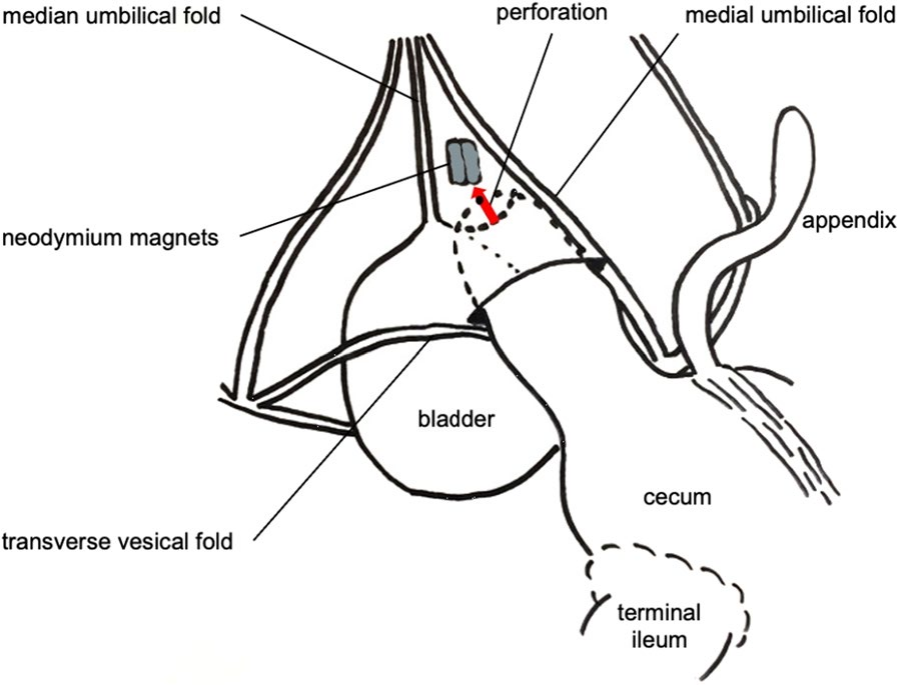
Anatomical schema (hypothesis). Although the precise mechanism of how this extremely rare internal SH with NMs migration have occurred, speculatively, the NM in his cecum was speculated strongly drawn to the direction for the dorsal side to the bladder, and the cecum with NM was incarcerated into supravesical fossa, then internal SH has developed

Although the precise mechanism of how this extremely rare internal SH with NM ingestion occured, speculatively, the NMs in his cecum was strongly drawn to attracted some metal on the child’s body such as the buckle of the car seatbelt, and the cecum with the NMs was incarcerated into the supravesical fossa, which led to the development of an internal SH **(**Fig. 4**)**. To our knowledge, this is the first case in which an ingested NMs were removed from an internal SH. Further preparation and dissemination of knowledge to get parents’ attention to NM ingestion are required.

## Conclusions

We experienced an extremely rare case of a pediatric patient who swallowed multiple NMs that migrated into an internal SH. Although the minimally invasive removal with a laparoscopic technique was successful after careful observation, early surgical intervention might be desirable.

## Data Availability

Not applicable.

## Abbreviations

FB: Foreign body
SH: Supravesical hernia
NM: Neodymium magnet
CT: Computed tomography
TAPP: Transabdominal pre-peritoneal
